# Influence of Ti_3_C_2_T_x_ MXene and Surface-Modified Ti_3_C_2_T_x_ MXene Addition on Microstructure and Mechanical Properties of Silicon Carbide Composites Sintered via Spark Plasma Sintering Method

**DOI:** 10.3390/ma14133558

**Published:** 2021-06-25

**Authors:** Mateusz Petrus, Jarosław Woźniak, Tomasz Cygan, Artur Lachowski, Dorota Moszczyńska, Bogusława Adamczyk-Cieślak, Anita Rozmysłowska-Wojciechowska, Tomasz Wojciechowski, Wanda Ziemkowska, Agnieszka Jastrzębska, Andrzej Olszyna

**Affiliations:** 1 Faculty of Material Science and Engineering, Warsaw University of Technology, Woloska 141 St., 02-507 Warsaw, Poland; jaroslaw.wozniak@pw.edu.pl (J.W.); tomasz.cygan@pw.edu.pl (T.C.); dorota.moszczynska@pw.edu.pl (D.M.); boguslawa.cieslak@pw.edu.pl (B.A.-C.); anita.wojciechowska.dokt@pw.edu.pl (A.R.-W.); agnieszka.jastrzebska@pw.edu.pl (A.J.); andrzej.olszyna@pw.edu.pl (A.O.); 2 Institute of High Pressure Physics, Polish Academy of Sciences, Sokolowska 29/37 St., 01-142 Warsaw, Poland; artur@unipress.waw.pl; 3 Faculty of Chemistry, Warsaw University of Technology, Noakowskiego 3 St., 00-664 Warsaw, Poland; wojciechowski16@gmail.com (T.W.); ziemk@ch.pw.edu.pl (W.Z.)

**Keywords:** composites, silicon carbide, CMCs, MXene, Ti_3_C_2_T_x_, sol-gel method, mechanical properties

## Abstract

This article presents new findings related to the problem of the introduction of MXene phases into the silicon carbide matrix. The addition of MXene phases, as shown by the latest research, can significantly improve the mechanical properties of silicon carbide, including fracture toughness. Low fracture toughness is one of the main disadvantages that significantly limit its use. As a part of the experiment, two series of composites were produced with the addition of 2D-Ti_3_C_2_T_x_ MXene and 2D-Ti_3_C_2_T_x_ surface-modified MXene with the use of the sol-gel method with a mixture of Y_2_O_3_/Al_2_O_3_ oxides. The composites were obtained with the powder metallurgy technique and sintered with the Spark Plasma Sintering method at 1900 °C. The effect adding MXene phases had on the mechanical properties and microstructure of the produced sinters was investigated. Moreover, the influence of the performed surface modification on changes in the properties of the produced composites was determined. The analysis of the obtained results showed that during sintering, the MXene phases oxidize with the formation of carbon flakes playing the role of reinforcement. The influence of the Y_2_O_3_/Al_2_O_3_ layer on the structure of carbon flakes and the higher quality of the interface was also demonstrated. This was reflected in the higher mechanical properties of composites with the addition of modified Ti_3_C_2_T_x_. Composites with 1 wt.% addition of Ti_3_C_2_T_x_ M are characterized with a fracture toughness of 5 MPa × m^0.5^, which is over 50% higher than in the case of the reference sample and over 15% higher than for the composite with 2.5 wt.% addition of Ti_3_C_2_T_x_, which showed the highest fracture toughness in this series.

## 1. Introduction

Despite many advantages, such as high hardness, resistance to high temperatures, and high wear resistance to friction, silicon carbide is used in industry to a limited extent [1,2,3,4]. Its main limitation is the low fracture toughness of carbide ceramics, which is 10 times lower than for aluminum and nearly 60 times lower than for maraging steel [5,6]. In order to increase the fracture resistance of silicon carbide and other ceramics, apart from the development of modern sintering technologies (spark plasma sintering, cold sintering process) [7,8], research is focused on the development of silicon carbide composites. There are a few materials that can be used as reinforcements in silicon carbide matrix composites, which at the same time improve fracture toughness without reducing other properties, such as hardness or thermal stability, e.g., SiC-carbon fiber composites or SiC/SiC composites [9,10,11]. The progress in the methods of manufacturing two-dimensional (2D) materials, initiated by obtaining graphene by Geim and Novoselov [12], created an opportunity for the development of a new family of composites reinforced with 2D crystals. The use of crystals with a 2D structure, such as graphene, graphene oxide, or surface-modified graphene as a reinforcement, by significantly reducing the growth of grain and introducing strengthening mechanisms into the matrix, causes a significant improvement of mechanical, electrical, and functional properties [13,14,15,16] with just a small addition of crystals with a 2D structure.

MXene phases are a new, extremely promising, and rapidly developing group of materials with a 2D structure, developed in 2011 by M. Naguib, which can be defined as two-dimensional (2D) transition metal carbides and nitrides [17,18,19]. Their unique geometry and stoichiometry, as well as hydrophilicity and a combination of mechanical, electrical, and chemical properties, were the reason for them to quickly find an application as reinforcement in polymer and metallic composites [20,21,22]. MXene based composites have already been used in many fields such as biotechnology, medicine, energy storage devices [23,24,25,26]. There is, however, not enough data on the use of MXene phases as reinforcement in ceramic composites. Their low thermal stability is the main reason for that fact, as they decompose in an oxygen-rich atmosphere above 300 °C [27].

Despite these difficulties, there are several papers describing successful attempts to obtain composites based on a ceramic matrix. Fei et al. [28] described an attempt to obtain MXene reinforced alumina matrix composites, which resulted in an increase in hardness and fracture toughness by nearly 300%. Guo et al. [29], in turn, proposed the unconventional cold isostatic pressure method, which allowed to sinter Zno-Ti_3_C_2_ composites at 300 °C to 92–92% of the theoretical density. They noted an increase in hardness of nearly 150% for 5 wt.% Ti_3_C_2_ addition, compared to unreinforced zinc oxide. In our previous work, we presented a method of obtaining composites based on silicon carbide reinforced with the Ti_2_C MXene phase [30]. Owing to the use of the modern spark plasma sintering technology, we obtained composites with a relative density close to theoretical, which were characterized by hardness and fracture toughness increased by 18 and 55%, respectively, for just 2% of the additive. The microscopic observations confirmed the presence of Ti_2_C phase flakes in the microstructure at the grain boundaries. This increase was possible thanks to the introduction of strengthening mechanisms such as crack bridging, crack deflection, and pull-outs in the material. According to the proposed model, an important aspect, in this case, is the presence of non-delaminated and agglomerated flakes of the MXene phases, especially for higher proportions of the Ti_2_C phase (above 2 wt.%). The same authors studied the influence of the Ti_3_C_2_ MXene phase on silicon nitride matrix composites [31]. The influence of the Ti_3_C_2_ MXene phase on the phase composition and the mechanical properties of the obtained sinters were studied. However, the presence of MXene in the final microstructure has not been confirmed. In the research [32], the oxidation of MXene phases was used to obtain TiC/SiBCN composites. During heating, the titanium oxide, formed as a result of decomposition of Ti_3_C_2_T_x_, reacted with carbon to form in situ particles of TiC. An interesting experiment was described in work [33]. The authors compared the influence of the Ti_3_C_2_ MXene phase and the Ti_3_C_2_ phase sputter-coated with high-melting metals (titanium and molybdenum) on the microstructure and mechanical properties of alumina. They established that the presence of a metallic coating may notably change the form of degradation in the MXene phases. In our previous publication [34] on obtaining SiC-2D-Ti_3_C_2_ MXene, we analyzed the structure of graphite flakes observed in the final microstructure of the obtained composites. The studies have shown that the MXene phases oxidize during sintering with the creation of highly defected carbon structures playing the role of reinforcement. The proposed explanation presents a mechanism in which, despite the application of vacuum, there are oxidizing factors present in the spaces between particles of the sintered material during the process of sintering that may lead to “pulling out” titanium atoms from the MXene structure, creating carbon flakes.

In the light of the above facts, it can be concluded that the basic aspects of ceramic-MXene composites are their thermal stability, tendency to agglomerate, and high impact of the surface condition of MXene flakes on their mechanical properties, and thus on the mechanical properties of the final composites. In this article, we present a proposal for the production of silicon carbide matrix composites reinforced with unmodified and surface-modified MXene phases. The MXene phases were obtained through the digestion and delamination process of the MAX Phases. MAX phases have been synthesized from pure elements using spark plasma sintering technology (SPS). Subsequently, a portion of the MXene phases was subjected to a modification process to form a Y_2_O_3_/Al_2_O_3_ oxide coating on its surface. The MXene phases prepared this way were then used to produce a series of SiC-MXene and SiC-MXene/Al_2_O_3_/Y_2_O_3_ composites. The effect of the addition of Ti_3_C_2_ MXene and Ti_3_C_2_ MXene/Y_2_O_3_/Al_2_O_3_ phases on the mechanical properties and the impact the surface modification of the strengthening phase has on the final properties of the composites were investigated.

## 2. Materials and Methods

### 2.1. Substrates

The following commercial powders were used to prepare a Ti_3_AlC_2_ MAX phase: pure titanium powder (GoodFellow, Cambridge, UK) with a chemical purity of 99.6% and an average particle size below 20 μm, pure aluminum powder with a chemical purity of 99.7% and synthetic graphite powder (Sigma-Aldrich, St. Louis, MO, USA) with a chemical purity of 99.9% and the average particle size below 20 μm. As a matrix, commercial β-SiC powder was used (Alfa Aesar, Ward Hill, MA, USA) with a chemical purity of 99.8% and 0.42 µm average particle size. Carbon black (Sigma-Aldrich, St. Louis, MO, USA) with a chemical purity of 99% and the average particle size below 100 nm and amorphous boron powder (International Enzymes Limited, Hampshire, UK) with a chemical purity of 96% and the average particle size 0.39 µm were applied as a sintering activators.

### 2.2. Preparation of the Ti_3_AlC_2_ MAX Phase

The first stage of obtaining the MXene phase is the synthesis of the Ti_3_AlC_2_ MAX phase. For this purpose, the titanium, aluminum, and carbon powders were wet blended in isopropyl alcohol (no. 1759, Stanlab, Lublin, Poland) using a ball-type mill (Fritsch Pulverisette, Fritsch, Idar-Oberstein, Germany), dried and sieved (# = 300 µm). The molar ratio of Ti:Al:C = 3:1:1.9 was applied. The SPS technique (FCT Systeme GMBH, Effelder-Rauenstein, Germany) was used in the reactive synthesis process. The Ti_3_AlC_2_ MAX phases were synthesized in the following parameters: temperature: 1300 °C, heating rate: 250 °C/min, vacuum atmosphere (*p* = 5 × 10^−1^ mbar). The obtained MAX phase was ground with the use of an automatic mortar grinder (Retsch KM100, Retsch GmbH, Haan, Germany) below 45 µm (grinding bowl speed = 70 rpm, applied force 5–12.5 daN).

### 2.3. Preparation of the Ti_3_C_2_ MXene Phase and Surface-Modified Ti_3_C_2_ MXene Phase

The Ti_3_C_2_T_x_ MXene phase was obtained by etching the Ti_3_AlC_2_ MAX phase with concentrated 48% hydrofluoric acid solution (no. 695068, Sigma-Aldrich, Taufkirchen, Germany). The ratio of the acid used in the process was 10 cm^3^ per 1 g of powder. This process was performed in a Teflon vessel, to which a 48% aqueous HF solution was added. The system was continuously stirred with the use of a magnetic stirrer (285-MS11H, Wigo, Pruszków, Poland) at 250 rpm. Then, the MAX phase powder was gradually added to it. The etching process was carried out for 24 h at room temperature with constant stirring. Following that, the mixture was left without agitation for sedimentation. The supernatant obtained was discarded, and the remaining Ti_3_C_2_T_x_ phase was washed with deionized water until the pH was neutral. The resulting MXene phase was then dried for 24 h at room temperature.

The Ti_3_C_2_ phase was surface-modified with a 1:1 layer composed of a mixture of 3% Y_2_O_3_ and 3% Al_2_O_3_. The process of modification was carried out using the sol-gel method. The precursor reagents, that is i-propoxide (ABCR, Karlsruhe, Germany) for yttrium (III) and isopropoxide (Sigma-Aldrich, Taufkirchen, Germany) for aluminum, were mixed in isopropanol to complete solubilization. Subsequently, Ti_3_C_2_ was immersed in the obtained reactants mixture, which was then continuously stirred at 250 rpm for 24 h at room temperature, and stored in a fume hood. Subsequently, the obtained Ti_3_C_2_/Y_2_O_3_/Al_2_O_3_ (denoted) was sedimented, washed with isopropanol, dried overnight, and then subjected to composites preparation.

### 2.4. Preparation of the Composites

The composites were prepared with the powder metallurgy technique and sintered with the use of the SPS method. Firs, the SiC-x wt.% Ti_3_C_2_T_X_ and SiC-x wt.% Ti_3_C_2_T_x_M powder mixtures (where x = 0.2, 0.5, 0.7, 1, 1.5, 2, 2.5, 3 wt.%) were wet blended for 8 h using a planetary ball-type mill. Propan-2-ol and alumina grinding balls (Nikkato, Osaka, Japan) were applied in this step. After drying, the powders were manually sieved (# = 300 µm). Composites were sintered with the use of spark plasma sintering. The parameters of the process were as follows: sintering temperature: 1900 °C, heating and cooling rate: 250 °C/min, 30 min dwell time, 50 MPa applied pressure, and vacuum. Based on the previous optimization work [35], the amount of sintering aids, i.e., boron and carbon, was 0.3 wt.% and 0.5 wt.%, respectively. As a reference sample, silicon carbide sinters with the addition of 0.3 wt.% of boron and 0.5 wt.% of carbon black were sintered at the same conditions.

### 2.5. Characterization of MAX/MXene Phases

The surface morphology and the shape of particles of the Ti_3_AlC_2_ MAX phase and Ti_3_C_2_ MXene 2D sheets were investigated with the use of an SEM, LEO 1530 (Zeiss, Maple Grove, MN, USA) scanning electron microscope. The phase composition of the obtained MAX phase was investigated with the use of X-ray diffraction (XRD, D8 ADVANCE, Bruker, Billerica, MA, USA), using CuKα radiation at a wavelength λ = 0.154056 nm. The parameters of this test were as follows: voltage: 40 kV, current: 40 mA, angular range: 10–90°, 0.05° step. The optical absorption spectra of the 2D sheets of Ti_3_C_2_T_x_ unmodified and modified with 3% wt. Y_2_O_3_ were analyzed using a UV-Vis spectrometer (Evolution 220, Thermo Scientific, Waltham, MA, USA) in distilled water and in isopropanol. Spectra were recorded in the range of 220–1100 nm. The measurement parameters were: scanning speed of 200 nm min^−1^, integration time of 0.30 s, resolution of 1.00 nm. Fourier transform infrared spectroscopy (FTIR) measurements were performed using FT-IR Nicolet iS5 from Thermo Electron, Waltham, MA, USA), equipped with a diffuse reflectance infrared Fourier transform (DRIFT) accessory. For the DRIFT measurements, the samples were mixed with dried KBr (Fluka, assay ≥ 99.5%) at a concentration of 2.5% wt. The resolution of the FTIR analyzer on which the measurements were performed was 2 cm^−1^. The spectra were recorded in the range of 4000–400 cm^−1^. Each spectrum was reported in an average of 30 scans. The zeta potential and measurements were performed using a NANO ZS ZEN3500 analyzer (Malvern Instruments, Malvern, UK) equipped with a back-scattered light detector operating at a 173° angle. The studies were carried out at 25 °C in distilled water and in isopropanol. The concentration of the studied samples was 5 × 10^−4^ g/cm^3^, and each sample was homogenized for 30 sec using mild sonication (Emmi H60, Emag, Salah, Germany). The zeta potential measurements were repeated 20 times. The results were expressed as the mean value of zeta potential ± SD.

### 2.6. Characterization of Composites

The density of the specimens was examined using the Archimedes method (PN-EN 1094-4:1998 Standard [36]). Hardness was measured with the Vickers Hardness Tester (FV-700e, Future-Tech, Kawasaki, Japan) using the indentation method under the load of 49.05 N. The fracture toughness of the produced composites was determined with the Vickers indentation crack length method under the load of 49.05 N. The Niihara, Morena, Hasselman equation was used. A total of 20 hardness measurements and 12 crack length measurements were performed for each sample. The phase composition of composites was analyzed with X-ray diffraction (XRD, D8 ADVANCE, Bruker, Billerica, MA, USA), using CuKα radiation at a wavelength of λ = 0.154056 nm. The parameters of this test were as follows: voltage: 40 kV, current: 40 mA, angular range: 20–100° with step 0.05°. The microstructure observations were performed on a scanning electron microscope (SEM Hitachi 5500, Hitachi, Tokyo, Japan). The observations were carried out at a 20 kV accelerating voltage. The scanning transmission electron microscopy images and diffraction patterns were obtained on an FEI Tecnai G2 F20 S-TWIN microscope (FEI, Hillsboro, OR, USA), operating at 200 kV and equipped with a Fischione 3000 high angle annular dark-field (HAADF) STEM detector.

## 3. Results

**Figure 1:**
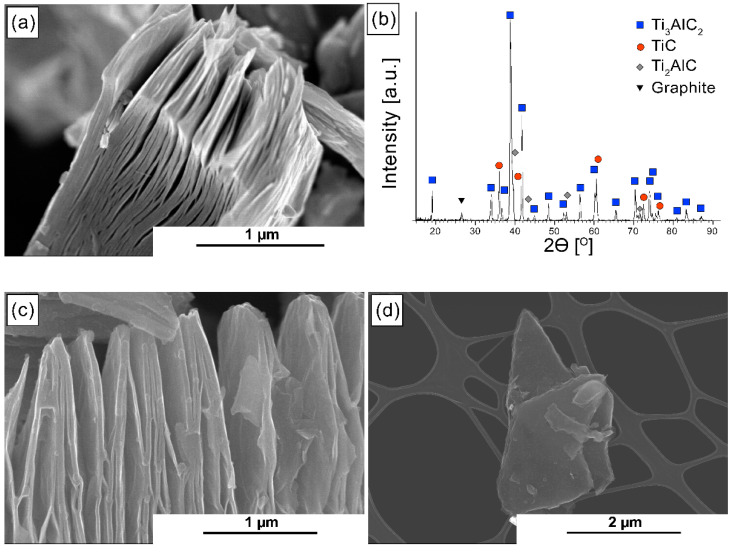

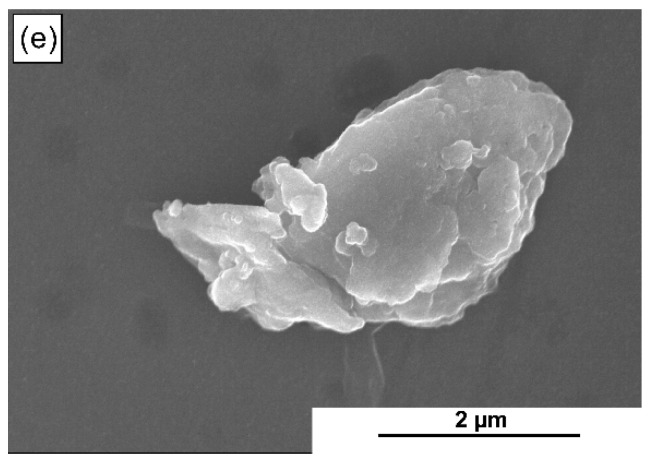
(**a**) Morphology of the obtained MAX phase; (**b**) XRD analysis of the obtained MAX phase; (**c**) morphology of the expanded Ti_3_C_2_ MXene; (**d**) morphology of 2D Ti_3_C_2_T_x_ crystals; (**e**) morphology of 2D Ti_3_C_2_T_x_ M crystals.

Figure 1a shows the morphology of the obtained MAX phase. The layered structure, characteristic of this group of materials, can be observed. The phase composition analysis, presented in Figure 1b, exhibited the presence of the Ti_3_AlC_2_ phase and small amounts of TiC, Ti_2_AlC, and graphite. Due to the presence of the Ti_3_AlC_2_ phase, the powders were subjected to selective etching. This way, expanded Ti_3_C_2_T_x_ MXene phases were obtained (Figure 1c), which were then subjected to delamination and surface modification. The 2D-Ti_3_C_2_ MXene phase (Figure 1d) and the 2D-Ti_3_C_2_ phase after the surface modification process (Figure 1e) can be observed. Both powders, before and after surface modification, are characterized by a flakes structure that shows many similarities to graphene.

**Figure 2:**
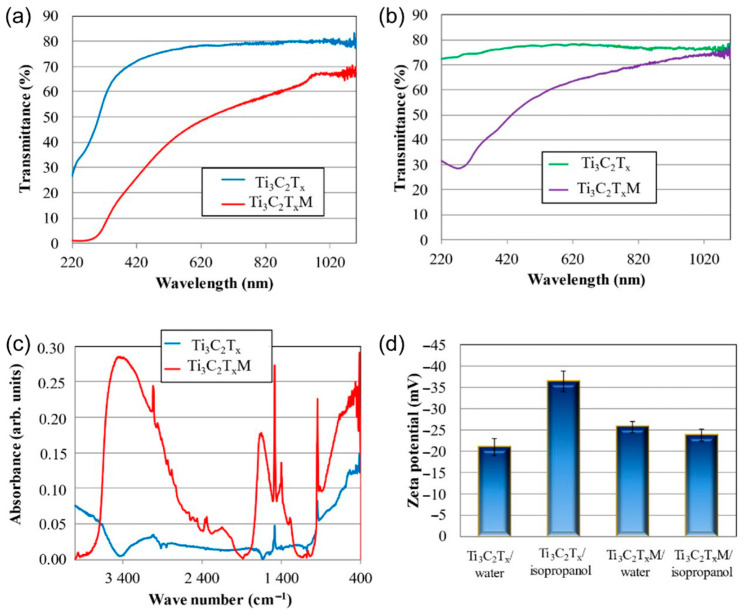
Ultraviolet visible spectrum (UV-VIS) of (**a**) Ti_3_C_2_T_x_ and Ti_3_C_2_T_x_M in distilled water, (**b**) Ti_3_C_2_T_x_ and Ti_3_C_2_T_x_M in isopropanol; (**c**) Fourier transform infrared spectroscopy (FTIR) spectrum of Ti_3_C_2_T_x_ and Ti_3_C_2_T_x_M crystals; (**d**) zeta potential of Ti_3_C_2_T_x_ and Ti_3_C_2_T_x_M crystals in different solutions.

When analyzing the UV-Vis spectrum of pristine 2D-Ti_3_C_2_T_x_ presented in Figure 2a, a significantly higher absorption peak, close to 270 nm, may be observed. It can be concluded that it is caused by the presence of TiO_2_ or Ti_2_O_3_ (or both) on the MXene phase surface. As it may be noticed both in the case of measurements in distilled water (Figure 2a) and in isopropanol (Figure 2b), the Ti_3_C_2_Tx phase exhibited a higher ability to absorb visible light over the entire range of the tested wavelength compared to the Ti_3_C_2_T_X_M phase. FTIR spectrum was performed for the pristine Ti_3_C_2_T_x_ MXene phase, and the phase was modified with 3 wt.% Y_2_O_3_. The results are presented in Figure 2c. As it may be observed, in the spectrum from the pristine Ti_3_C_2_T_x_ MXene phase, signals at 3013 and 1487 cm^−1^ coming from the C-H bonds are present. There is also a signal at 948 cm^−1^ coming from the C-F functional group, which was confirmed in our previous work [37]. In the spectrum of the Ti_3_C_2_T_X_ MXene phase modified with 3 wt.% Y_2_O_3_/Al_2_O_3_, a wide band can be observed within the range of 3500–2900 cm^−1^ derived from the stretching vibrations of the hydroxyl groups -O-H [38,39], which are directly bound to titanium atoms on the surface of the MXene phases and come from the substrates used for the synthesis of Y_2_O_3_. The peaks at 3018 cm^−1^ and 1487 cm^−1^ come from C-H bonds, and 1653 cm^−1^ come from C = O bonds. There is also a peak at 948 cm^−1^ in the spectrum, which comes from the C-F bonds. The lower side of the wavenumber spectrum shows three sharp peaks at 420, 471, and 512 cm^−1^ that are attributed to the Y-O bond from Y_2_O_3_ [40]. The zeta potential measurements are presented in Figure 2d. The surface modification results in higher stability of 2D Ti_3_C_2_T_X_ flakes in an aqueous solution and lower in isopropanol, whereas in the case of the 2D Ti_3_C_2_T_x_ phase not subjected to surface modification, the stability in an isopropanol solution is higher.

**Figure 3:**
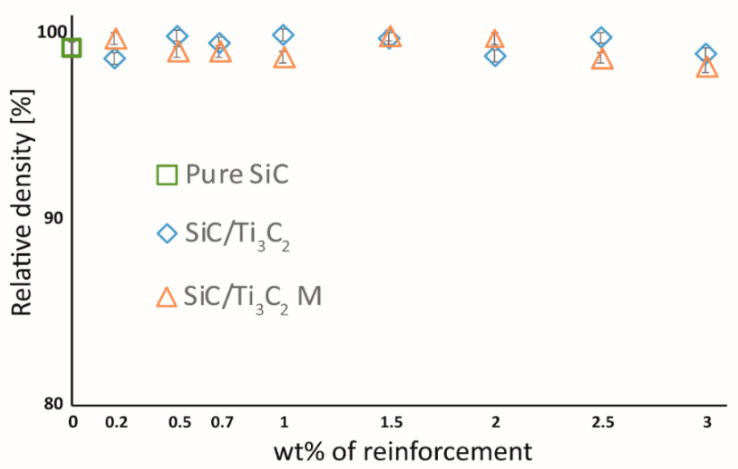
The relative density of obtained composites.

**Figure 4:**
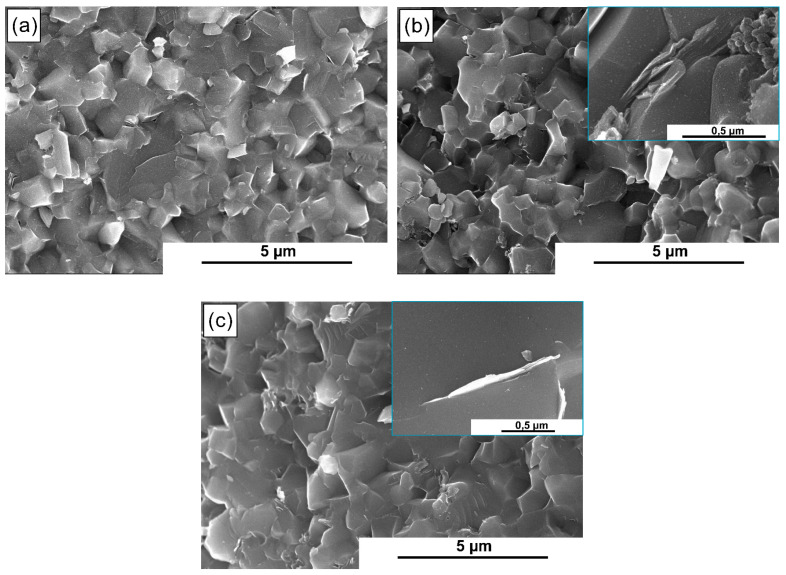
Scanning electron microscope image of the fracture surface of (**a**) pure SiC sinter, (**b**) SiC + 1.5 wt.% Ti_3_C_2_T_x,_ and (**c**) SiC + 1.5 wt.% Ti_3_C_2_T_x_M.

The relative density of the obtained composites was presented in Figure 3. All of the produced composites, regardless of the type of reinforcement used, are characterized by relative density oscillating between 98% and 99.5%. Taking into account the error bars, no significant influence of the addition of MXene phases and their amount on the sinter density can be found. For the purpose of determining the influence of MXene phase addition on the microstructure, the fracture surfaces of the obtained composites were subjected to microscopic observations. The results of these observations are presented in Figure 4. All of the obtained composites, similarly to the reference sample (Figure 4a), present a homogeneous, fine-crystalline microstructure. The presence of flakes phase on the grain boundaries, which morphology corresponds to one of MXene phases, is noticeable both in the case of composites with the Ti_3_C_2_T_x_ phase addition (Figure 4b) and for composites with the Ti_3_C_2_T_X_M phase addition (Figure 4c). For composites having the addition of MXene phases where surface was not modified, the flakes in the reinforcement phase are of significantly higher thickness than the flakes in the Ti_3_C_2_T_X_M phase.

**Figure 5:**
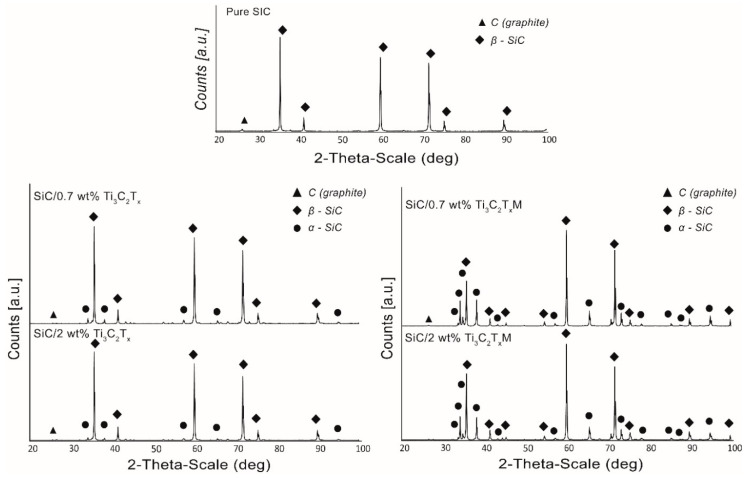
Phase composition of the obtained composites.

In order to determine the influence of the used MXene phases on the phase composition of composites, the reference sample, and composites with the addition of 0.7 wt.% and 2 wt.% Ti_3_C_2_ and Ti_3_C_2_M were analyzed. The results are presented in Figure 5. In the case of the reference sample, only one β-SiC phase and a small amount of carbon were confirmed. In the case of composites with the addition of the Ti_3_C_2_T_X_ phase, apart from β-SiC, the presence of the α-SiC phase was found. However, these peaks are small, indicating the presence of a negligible amount of additional phase. The situation is slightly different in the case of composites with the addition of Ti_3_C_2_T_X_M. In this case, the presence of the α-SiC phase was also confirmed, but the higher intensity of the peaks suggests that the process of the β-SiC → α-SiC phase transformation occurred with higher intensity. It is also worth noting that the influence of the amount of the strengthening phase on the composite’s phase composition and the amount of the α-SiC phase formed is not observed.

The influence of MXene phases on the mechanical properties is presented in Table 1. Both in the case of Ti_3_C_2_T_X_ and Ti_3_C_2_T_X_M addition, a small addition of the reinforcement phase is enough to cause a major increase in toughness. For composites reinforced with the Ti_3_C_2_T_X_ phase, the highest toughness was observed for 1.5 wt.% (almost 15% when compared to the reference sample), whereas for composites reinforced with the phase having a modified surface, the highest toughness was noted for the composite with 1 wt.% addition of the Ti_3_C_2_T_X_M phase. It is worth emphasizing that for the entire analyzed range, the composites with the addition of modified MXene phases present higher toughness than the ones reinforced with unmodified MXene phases. Moreover, all of the obtained composites are characterized with higher fracture toughness than the reference sample, though the differences between them are not evident. Both series present a similar course and the values of the VIF factor. The sample with 1 wt.% addition of Ti_3_C_2_T_X_M constitutes an exception, as it is characterized with a fracture toughness of 5 MPa × m^0.5^, which is over 50% higher than in the case of the reference sample and over 15% higher than for the composite with 2.5 wt.% addition of Ti_3_C_2_T_X_, which showed the highest fracture toughness in this series.

**Figure 6:**
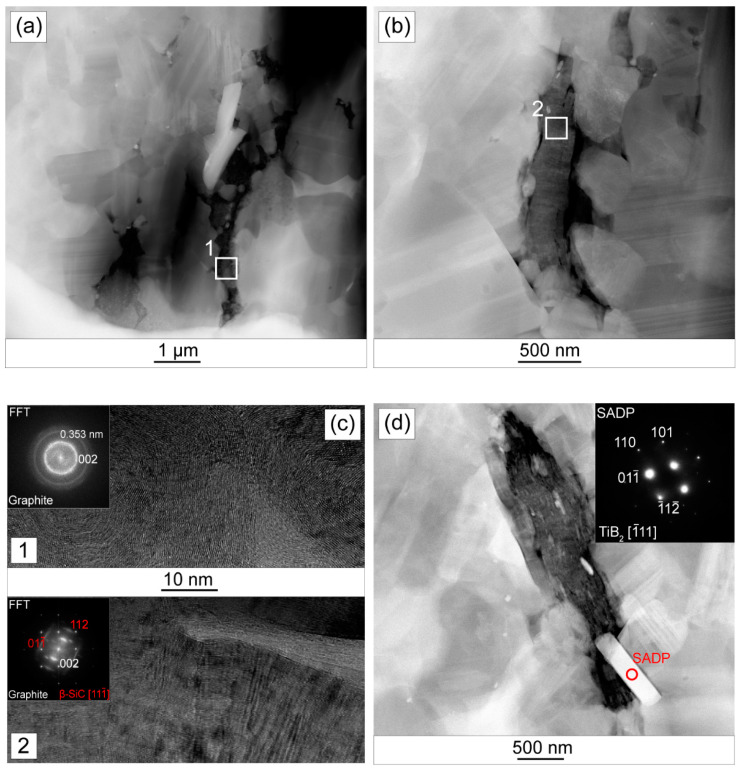
TEM analysis of SiC-2 wt.% Ti_3_C_2_T_x_ composites (**a**) HAADF scanning transmission electron microscope (STEM) image of reinforcing phase; (**b**) HAADF scanning transmission electron microscope (STEM) image of the reinforcing phase; (**c**) HRTEM image of area 1 and 2 with the Fourier transform; (**d**) HAADF scanning transmission electron microscope (STEM) image of the observed particles with the electron diffraction.

The results of TEM observations for SiC + 2 wt.% Ti_3_C_2_T_x_ are presented in Figure 6. The presence of two types of flake structures was confirmed. The first type has the form of irregular clusters of lamellar structures stretched along the grain boundaries for about several microns of length (Figure 6a). The second type, on the other hand, forms more compact structures, corresponding to the morphology of the MXene phase (Figure 6b). Defects of the reinforcing phase-matrix interface in the form of pores, voids, and discontinuities were found around both types of structures. In addition, both types of structures in the high angle annular dark-field mode (HAADF) show much lower intensity compared to the matrix grains. The intensity of the HAADF signal is roughly proportional to the square of the average atomic number (Z^2^) of the tested area; hence, higher intensity of the grains in the HAADF image suggests that they consist of heavier elements than the reinforcement phase. The high-resolution transmission electron microscopy (HRTEM) images and the Fourier transform from these areas confirm the above assumption (Figure 6c). The obtained diffraction images indicate that the observed flake structures consist of carbon. In the case of the first type of structures, the presence of diffraction rings was detected, which in combination with the HRTEM image (Figure 6c, area 1), indicates their strong defect and a high degree of amorphousness. The following rings correspond to the interplanar distances: 0.350–0.210–0.173–0.122 nm, which corresponds to the graphite planes (002)–(100)–(004)–(110) [41]. The second type of observed structure (Figure 6c, area 2) is, in turn, characterized by a significantly smaller defect, as evidenced by the obtained diffraction pattern. It also confirms that these structures are made of graphite. The analysis of the microstructures also revealed the presence of elongated particles adhering to the flakes’ structures (Figure 6d). Their much higher intensity in the HAADF mode suggests that their chemical composition differs from the matrix. Electron diffraction identified them as TiB_2_ particles.

**Figure 7:**
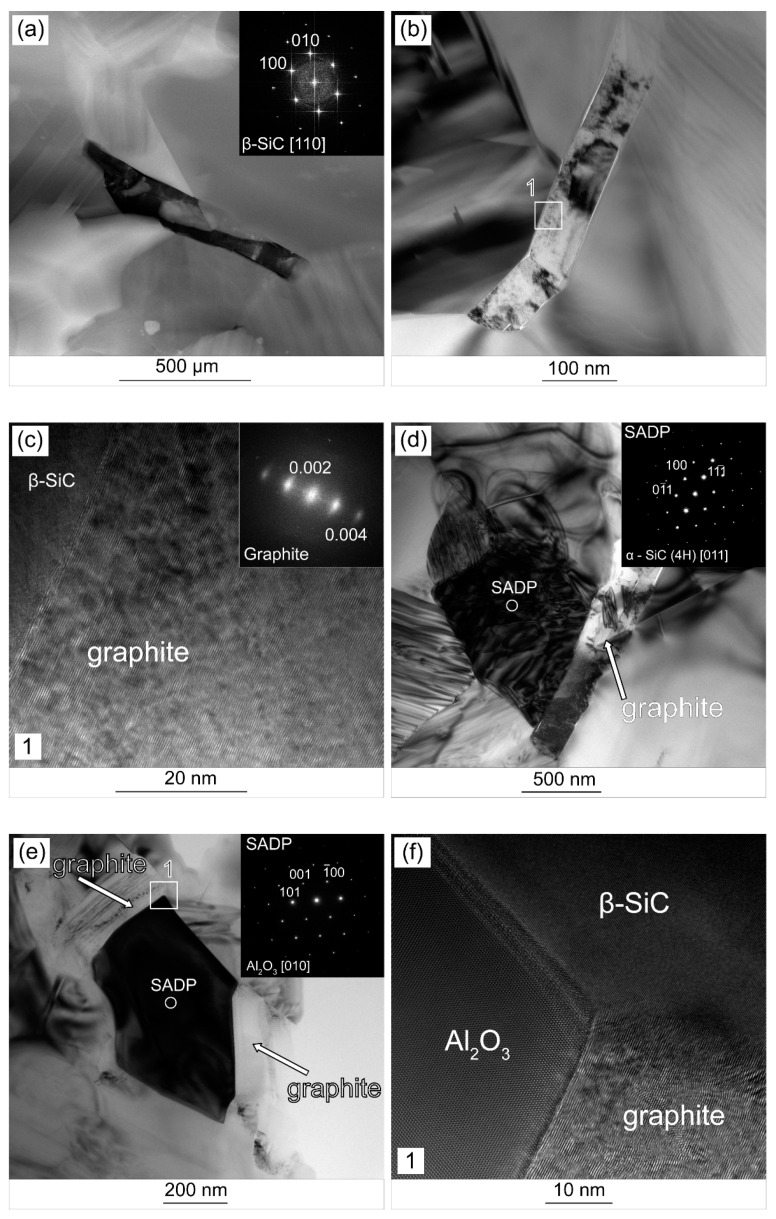
TEM analysis of SiC-2 wt.% Ti_3_C_2_T_x_M composites (**a**) HAADF STEM image of reinforcing phase and electron diffraction; (**b**) TEM BF image of the reinforcing phase; (**c**) HRTEM image of area 1 and the Fourier transform; (**d**) TEM BF image of the composites and selected area diffraction pattern (SADP); (**e**) TEM BF image of graphite structures surrounding the Al_2_O_3_ grain; (**f**) HRTEM image of the interface boundary between graphite, SiC and Al_2_O_3_ grains.

Different types of structures were observed in composites with the addition of surface-modified MXene phases. They show a compact structure and a much better interface without pores, voids, and discontinuities. (Figure 7a,b). Their intensity in the HAADF mode also indicates that they are made of lighter elements than the matrix. The obtained electron diffraction confirmed that they are made of graphite (Figure 7c). In this case, no bending of atomic planes or defects characteristic for the structures in the composites shown in Figure 6 were observed. The presence of α-SiC phase grains was also detected, which confirms the results of the phase composition analysis (Figure 7d). The analysis also allowed us to identify single Al_2_O_3_ grains (Figure 7e). They are located directly in the vicinity of the flake carbon structures. In the high-resolution mode, the high quality of the interfacial boundaries can be seen (Figure 7f). The atomic planes of one material smoothly transfer into the planes of the other. There are no visible defects (pores, voids) observed in composites with the addition of non-surface-modified Ti_3_C_2_T_x_ phases. In both cases, the presence of flake structures in the microstructure that could be identified as Ti_3_C_2_T_x_ phases was not confirmed.

## 4. Discussion

The analysis of the obtained results clearly suggests that the addition of MXene phases to the silicon carbide matrix improves the mechanical properties. However, detailed analysis of the produced composites microstructures did not confirm the presence of MXene phases, and all of the observed flake structures were identified as flakes of more or less defective graphite. In their immediate vicinity, TiB_2_ grains were also identified in the case of SiC-Ti_3_C_2_T_x_ composites and Al_2_O_3_ grains in the case of SiC-Ti_3_C_2_T_x_M. Their presence indicates the origin of carbon flakes and suggests that they are the result of MXene phase decomposition under the influence of sintering conditions.

The presence of carbon structures in the final microstructure was also confirmed in our previous publication [34]. According to the proposed mechanism, during the process of sintering, the spaces between grains located in close vicinity to MXene phases are enriched in oxidizing factors due to the release of physically absorbed water, oxygen, and functional groups (-OH, -O) from the surface. Their presence causes the oxidation of MXene phases, as suggested by Naguib et al. [42]. As a result, we obtained carbon structures and titanium oxide, which, in the temperature of sintering, transformed into a liquid phase distributed on the grain boundaries. The presence of the liquid during the sintering process is supported by the phase transformation of β-SiC → α-SiC observed in both series of tested composites in the presented experiment. It also intensifies the diffusion processes responsible for the rate of phase transformation [43].

The presence of such structures at the grain boundaries will, as in the case of ceramic composites reinforced with graphene materials, introduce mechanisms responsible for the improvement of mechanical properties into the matrix [44]. The observed changes in hardness and fracture toughness of the produced composites, i.e., their increase with an increase in the amount of the strengthening phase, and then, after reaching the maximum value, a slow decrease, is characteristic for ceramic composites reinforced with graphene. A similar course of hardness and fracture toughness as a function of Ti_2_C MXene phase addition for composites based on silicon carbide was recorded in our previous work [30]. A slight decrease in the average grain size of the carbide matrix was confirmed even with a small addition of the MXene phase, as well as the presence of mechanisms increasing fracture toughness, such as delamination, deflection, and crack branching. Due to the consumption of propagating crack energy, these are the mechanisms effectively increasing fracture toughness [45,46]. The increase in fracture toughness observed in the case of composites obtained in this study confirms the presence of the mentioned mechanisms.

It is also extremely interesting to compare the two series of composites with each other, indicating the observed differences. Detailed observations of the obtained composites’ microstructure showed that Ti_3_C_2_T_X_ phases with modified surfaces exhibit a significantly lower tendency to agglomerate. If such tendency is lower, the number of filled grain boundaries in composites increases, whereas the weight percent of the reinforcement phase remains the same. This may be the reason for the greater hardness of composites with the addition of Ti_3_C_2_T_X_M phase and the greater standard deviation of single hardness results for composites with the addition of unmodified MXene phases. The differences in the mechanical properties of individual series may also result from the presence of an additional α-SiC phase in the SiC/Ti_3_C_2_T_X_M composites.

Another important factor that may cause higher hardness of SIC/Ti_3_C_2_T_X_M composites and their higher fracture toughness in the range of 1–2 wt.% may be significant differences in the structure of carbon flakes and the quality of the matrix-carbon flake interface (Figure 6 and Figure 7). In the case of composites with the addition of the unmodified Ti_3_C_2_T_X_ phase, the presence of two types of carbon structures was observed, some of which show a strong defect at the level of single atomic planes and show a certain degree of amorphism. The latter, in turn, are graphite structures with a more ordered structure. In addition, voids and discontinuities at the interface were observed around the strongly damaged structures, which may cause a decrease in fracture toughness [16]. In the case of SiC-Ti_3_C_2_T_X_M composites, graphite structures show a high degree of order, and the interface is devoid of these disadvantages. Based on these observations, it can be concluded that the surface modification of MXene phases may change the way of their thermal decomposition during sintering, which results in obtaining other structures.

The reason for this phenomenon may be a complex system of oxides around the Ti_3_C_2_T_X_ MXene flakes, namely Al_2_O_3_ and Y_2_O_3_ being part of the coating and TiO_2_ present on the Ti_3_C_2_T_X_ surface as a residue of the etching process [26]. If considering only two-component systems that can be formed in such an environment, both Al_2_O_3_-TiO_2_ and Y_2_O_3_-TiO_2_ can form a liquid phase at the sintering temperature [47]. The presence of an additional liquid phase is confirmed by a greater amount of α-SiC phase in the tested composites (Figure 5). Moreover, it is possible that in the vicinity of the liquid phase, the titanium atoms would diffuse easier and faster from the interior of the Ti_3_C_2_ phase [48,49], thus creating the observed carbon flake structures. Their faster creation could provide a greater amount of time needed to organize the structure, which would explain the significant differences in the level of disorder. Moreover, the greater amount of liquid phase facilitates the sintering processes taking place around the strengthening phase, which may explain the better quality of the interface [50]. Due to the possibility of a local disturbance of the composition favoring the formation of the glassy phase, oxide particles will remain around some carbon flakes, which can be observed in Figure 7f.

When comparing the obtained results to the ones described in the previous work [30], it may be stated that the application of surface modification of the MXene phases allowed to obtain similar results of hardness and fracture toughness with a lower addition of the reinforcement phase, that is 2 wt.% for the Ti_3_C_2_T_X_ phase and 1 wt.% for the Ti_3_C_2_T_X_M phase. Due to the fact that the obtained carbon structures present morphological similarity to graphene, it seems justifiable to compare the obtained results to the silicon carbide matrix composites reinforced with graphene [51]. For 1 wt.% addition of multilayer flake graphene and with the use of the same method of sintering, the obtained hardness was at 26 GPa level, whereas fracture toughness amounted to 4.1 MPA × m^0.5^. The composite with 1 wt.% addition of the Ti_3_C_2_T_X_M phase is, admittedly, characterized with lower toughness (for almost 10%), though it presents over 20% higher fracture toughness.

## 5. Conclusions

The article presents the research aimed at determining the effect of the addition of the Ti_3_C_2_T_x_ MXene phase and the Ti_3_C_2_T_x_ phase surface modified with Y_2_O_3_ and Al_2_O_3_ oxides on the mechanical properties of silicon carbide. The performed observations confirm that the addition of a small amount of MXene phases to the ceramic matrix may increase the mechanical properties. A detailed analysis of the obtained microstructures also showed that during sintering, the Ti_3_C_2_Tx phase is oxidized, with the formation of flake carbon structures with a defective graphite structure. These structures play the role of strengthening, improving the fracture toughness, and increasing the hardness of the produced composites. The studies have also shown that it is possible to use the sol-gel method to create an oxide layer containing Al_2_O_3_/Y_2_O_3_ on the surface of MXene phases. The use of the MXene phase prepared in this way allows for obtaining composites with higher hardness and higher fracture toughness. The sample with 1 wt.% addition of Ti_3_C_2_T_x_M is characterized with a fracture toughness of 5 MPa × m^0.5^, which is over 50% higher than in the case of the reference sample and over 15% higher than for the composite with 2.5 wt.% addition of Ti_3_C_2_T_x_, which showed the highest fracture toughness in this series. The proposed explanation of the observed changes assumes the formation of a liquid phase, the presence of which improves the interface and probably affects the degree of damage of the formed carbon structures. This phenomenon is extremely interesting and requires further research and in-depth analysis in the context of the application potential of the surface-modified MXene phases.

## Figures and Tables

**Table 1:** Mechanical properties of the obtained composites.

Sample	Hardness (GPa)	Fracture Toughness (MPa × m^0.5^)
SiC	19.9 ± 0.78	3.31 ± 0.2
SiC/0.2 wt.% of Ti_3_C_2_T_x_	21.2 ± 0.88	3.54 ± 0.27
SiC/0.5 wt.% of Ti_3_C_2_T_x_	21.7 ± 0.85	3.95 ± 0.33
SiC/0.7 wt.% of Ti_3_C_2_T_x_	21.5 ± 0.98	3.94 ± 0.39
SiC/1 wt.% of Ti_3_C_2_T_x_	21.9 ± 0.95	3.92 ± 0.33
SiC/1.5 wt.% of Ti_3_C_2_T_x_	22.0 ± 0.84	4.00 ± 0.21
SiC/2 wt.% of Ti_3_C_2_T_x_	21.8 ± 0.89	3.83 ± 0.43
SiC/2.5 wt.% of Ti_3_C_2_T_x_	21.0 ± 0.87	4.33 ± 0.44
SiC/3 wt.% of Ti_3_C_2_T_x_	19.5 ± 1.0	4.23 ± 0.55
SiC/0.2 wt.% of Ti_3_C_2_T_x_M	22.6 ± 0.59	3.65 ± 0.24
SiC/0.5 wt.% of Ti_3_C_2_T_x_M	22.8 ± 0.57	3.78 ± 0.21
SiC/0.7 wt.% of Ti_3_C_2_T_x_M	22.7 ± 0.61	3.98 ± 0.17
SiC/1 wt.% of Ti_3_C_2_T_x_M	24.3 ± 0.61	5.00 ± 0.16
SiC/1.5 wt.% of Ti_3_C_2_T_x_M	23.6 ± 0.56	4.54 ± 0.17
SiC/2 wt.% of Ti_3_C_2_T_x_M	22.8 ± 0.43	4.21 ± 0.20
SiC/2.5 wt.% of Ti_3_C_2_T_x_M	22.0 ± 0.50	4.25 ± 0.21
SiC/3 wt.% of Ti_3_C_2_T_x_M	22.5 ± 0.60	3.89 ± 0.14

## Data Availability

The data presented in this study are available on request from the corresponding author.
